# Randomized Open Investigation Determining Steroid Dose in Severe COVID-19: The ROIDS-Dose Clinical Trial

**DOI:** 10.7759/cureus.31086

**Published:** 2022-11-04

**Authors:** Carlos X Rabascall, Becky X Lou, Sean Dhar, Zubair Hasan, Craig Fryman, Stephanie Izard, Mina Makaryus, Sudeep Acharya, Fiore Mastroianni, Martin Kamper, Sean Duenas, Jonathan Gong, Dimple Shah, Sameer Khanijo, Daniel Ying, Junaid Habibullah, Dae Hyeon Kim, Ryan Butzko, Margarita Oks, Brian Birnbaum, Jonathan Moore, Anup K Singh, Luis Quintero, Michael Lau, Jared Honigman, Ayelet Hilewitz, Kruti Shah, Joseph Simonson, Abhinav Agrawal, Matthew Frank, Adey Tsegaye, Mangala Narasimhan, Harly Greenberg, Stella S Hahn

**Affiliations:** 1 Internal Medicine/Pulmonary and Critical Care, Donald and Barbara Zucker School of Medicine at Hofstra/Northwell, Hempstead, USA; 2 Internal Medicine/Pulmonary and Critical Care, University of Kansas Medical Center, Kansas City, USA; 3 Internal Medicine/Pulmonary and Critical Care, Catholic Health Initiatives, St. Joseph Health Regional Hospital, Bryan, USA; 4 Institute of Health System Science, Feinstein Institutes for Medical Research, Northwell Health, Manhasset, USA; 5 Internal Medicine, Donald and Barbara Zucker School of Medicine at Hofstra/Northwell, Hempstead, USA; 6 Internal Medicine/Pulmonary and Critical Care, Jersey Shore University Medical Center, Neptune City, USA

**Keywords:** pneumonia, respiratory failure, coronavirus disease 2019, covid-19 respiratory failure, dexamethasone, steroids, sars-cov-2, covid-19

## Abstract

Introduction

Treatment with dexamethasone reduces mortality in patients with coronavirus disease 2019 (COVID-19) pneumonia requiring supplemental oxygen, but the optimal dose has not been determined.

Objective

To determine whether weight-based dexamethasone of 0.2 mg/kg is superior to 6 mg daily in reducing 28-day mortality in patients with COVID-19 and hypoxemia.

Materials and methods

A multicenter, open-label, randomized clinical trial was conducted between March 2021 and December 2021 at seven hospitals within Northwell Health. A total of 142 patients with confirmed COVID-19 and hypoxemia were included. Participants were randomized in a 1:1 ratio to dexamethasone 0.2 mg/kg intravenously daily (n = 70) or 6 mg daily (n = 72) for up to 10 days.

Results

There was no statistically significant difference in the primary outcome of 28-day all-cause mortality with deaths in 12 of 70 patients (17.14%) in the intervention group and 15 of 72 patients (20.83%) in the control group (p = 0.58). There were no statistically significant differences among the secondary outcomes.

Conclusion

In patients with COVID-19 and hypoxemia, the use of weight-based dexamethasone dosing was not superior to dexamethasone 6 mg in reducing all-cause mortality at 28 days.

Clinical trial registration

This study was registered under ClinicalTrials.gov (identifier: NCT04834375).

## Introduction

Treatment of coronavirus disease 2019 (COVID-19) patients with respiratory failure has been challenging and multiple studies have investigated therapies to mitigate the dysregulated immune responses that have been directly implicated in the severity of the illness associated with severe acute respiratory syndrome coronavirus 2 (SARS-CoV-2) [1-10]. Corticosteroids exert anti-inflammatory effects through different mechanisms and have been shown to reduce inflammation in a variety of lung disorders with the added benefit of being low-cost and widely available [11-13]. Early in the pandemic, the use of steroids was discouraged based on prior experience with severe acute respiratory syndrome coronavirus and Middle East respiratory syndrome [14]. Dexamethasone has since emerged as one of the few proven therapies for patients with COVID-19 pneumonia with hypoxemia.

The Randomised Evaluation of COVID-19 Therapy (RECOVERY) trial, the largest randomized control trial to date evaluating the role of steroids in COVID-19, showed that the use of dexamethasone 6 milligrams (mg) daily for up to 10 days was associated with a decrease in mortality in COVID-19 patients requiring oxygen supplementation, particularly in those requiring invasive mechanical ventilation (MV) [15]. Since these findings were reported, the use of dexamethasone has been recommended by the Infectious Diseases Society of America, the US National Institutes of Health, and the World Health Organization for the treatment of severe COVID-19 [16-18].

Several other trials have demonstrated the mortality benefit of steroids in severe COVID-19 [19-21]. However, there is significant heterogeneity regarding the dose and the type of corticosteroid used. A limited number of studies that compare different doses of dexamethasone in COVID-19 exist, but the results are conflicting. The COVID STEROID 2 trial compared the effects of 12 mg versus 6 mg of dexamethasone in patients with COVID-19 and hypoxemia but did not reveal a statistically significant difference in the number of days alive without life support between the two groups [22]. The HIGHLOWDEXA trial compared the standard 6 mg dose to a higher dexamethasone dose of 20 mg daily for five days followed by 10 mg daily for five days in patients with severe COVID-19 and showed a reduction in clinical deterioration at 11 days after randomization in the high dose group [23].

Despite the established success of dexamethasone, there is still uncertainty surrounding its optimal dosing. This question is especially important to answer as there still exists a relative paucity of proven, effective, and most importantly, affordable and accessible therapies in the fight against COVID-19. Therefore, it is essential that the current use of proven therapies, such as dexamethasone, be optimized. The prior trials comparing different steroid dosing had conflicting results. A weight-based approach to dexamethasone dosing has not been studied and may balance dose-dependent benefits and toxicities.

The Randomized Open Investigation Determining Steroid Dose in Severe COVID-19 (ROIDS-Dose) clinical trial was conducted to evaluate the efficacy and safety of a higher dexamethasone dose in hospitalized patients with COVID-19 and hypoxemia. We hypothesized that weight-based dosing of dexamethasone, equivalent to methylprednisolone 1 mg per kilogram (kg) per day, a dose routinely used to treat other inflammatory conditions of the lungs [24,25], compared to dexamethasone 6 mg, would reduce mortality in patients with COVID-19 and hypoxemia.

## Materials and methods

This trial was an investigator-initiated, multicenter, prospective, open-label, randomized, clinical trial that was conducted at seven hospitals in Northwell Health in New York, including Long Island Jewish Medical Center, North Shore University Hospital, Lenox Hill Hospital, South Shore University Hospital, Valley Stream Hospital, Plainview Hospital, and Mather Hospital. The study protocol was approved by the Northwell Health Institutional Review Board (IRB # 21-0171) and the Clinic Research Unit at the Feinstein Institutes for Medical Research. A data and safety monitoring board oversaw the safety of the trial participants and conducted three planned interim analyses. Written, verbal, or electronic informed consent was obtained from eligible patients or their legally authorized representatives prior to enrollment.

Patients underwent screening and randomization between March 19, 2021, and December 28, 2021. Eligible patients were adults ≥18 years of age with a positive SARS-CoV-2 polymerase chain reaction test who required oxygen supplementation or had a documented oxygen saturation of less than 94%. Exclusion criteria included patients who used corticosteroids for >48 hours within 15 days prior to enrollment, received a dose higher than dexamethasone 6 mg or its equivalent prior to enrollment, used immunosuppressive medications, were on chronic oxygen supplementation, had a known history of dexamethasone allergy, were pregnant, had advanced directives with “do not resuscitate” or “do not intubate” orders, and lacked capacity and did not have a legally authorized representative to provide informed consent.

Randomization was performed using a computer-generated allocation sequence via the Research Electronic Data Capture (REDCap) system [26,27]. Eligible patients were randomized in a 1:1 ratio stratified by the trial site to either the standard dose group or the intervention group.

The control group received dexamethasone 6 mg intravenously once daily, and the intervention group received dexamethasone 0.2 mg/kg intravenously once daily, with a maximum dose of 20 mg. An upper limit of 20 mg for the intervention group was selected based on prior studies [13,23] to minimize potential adverse effects associated with higher doses of steroids [12]. No patients received less than 6 mg daily. The duration of treatment in both arms was 10 days or until discharge, whichever came first. If treatment with dexamethasone for COVID-19 was initiated prior to enrollment, the intervention period was shortened so that no patients received dexamethasone for more than 10 days per the trial protocol. Individual dose modifications were not permitted unless the patient reached a principal safety endpoint. All other interventions were at the discretion of the clinicians.

Study data obtained were recorded in REDCap [26,27] by trained study personnel. All patients were followed during their hospitalization and up to 28 days after enrollment. If patients were discharged prior to 28 days, a follow-up survey was conducted by study personnel via e-mail or a telephone call. An interim safety and result analysis was performed after the first 20 patients were enrolled, and subsequent analyses occurred when 50 and 100 patients were recruited. A designated committee was assigned for this purpose.

The primary outcome was all-cause mortality at 28 days after enrollment. The secondary outcomes included escalation to higher modes of supplemental oxygen (venturi mask, non-rebreather mask, high-flow nasal cannula, or non-invasive ventilation); the need for intensive care unit (ICU) admission; escalation to MV; duration of MV; the need for extracorporeal membrane oxygenation (ECMO); need for tracheostomy; ICU length of stay; hospital length of stay; development of secondary bacterial or fungal infections confirmed by culture data; development of clinically significant hyperglycemia, defined as requiring ICU admission for treatment of hyperglycemia or requirement of an insulin infusion; disposition upon hospital discharge; and need for oxygen supplementation upon discharge.

Sample size calculation

The sample size calculation was based on the primary hypothesis that weight-based dexamethasone dosing would be associated with a decrease in mortality in COVID-19 patients requiring oxygen supplementation. In the RECOVERY trial, the incidence of death was found to be 23.3% for patients receiving dexamethasone among COVID-19 patients who received oxygen without MV [15]. Another study found that the incidence of death for COVID-19 patients was 5.9% for those receiving methylprednisolone [20]. This estimate was assumed to be similar to weight-based dosing of dexamethasone given that at the time of study design there were no clinical trials comparing different doses of dexamethasone.

Assuming the proportion of mortality for those receiving usual care (dexamethasone 6 mg) was 23.3% and the proportion of mortality for those receiving the intervention (dexamethasone weight-based at 0.2 mg/kg) was reduced to 5.9%, a sample size of 128 (64 per arm) would have had 80% power to detect a difference between the two arms using a two-group chi-square test with a 5% two-sided level of significance. To account for the expected attrition of approximately 10%, the investigators had an enrollment target of 142 patients in this study.

Study variables

Data on 143 patients were collected in REDCap [26,27]. One record was entered in error and was excluded for a new total of 142 patients. These 142 patients were randomized in a 1:1 ratio using REDCap [26,27] to receive either dexamethasone 0.2 mg/kg (treatment arm) or dexamethasone 6 mg (control arm). One withdrawn patient was included in the analysis when data were available.

The outcomes of all-cause mortality at 28-days post-enrollment, including ICU admission, higher oxygen supplementation, invasive MV, ECMO, tracheostomy, oxygen supplementation on discharge, secondary bacterial or fungal infection, and clinically significant hyperglycemia, were assessed as binary variables. Discharge disposition was collapsed to facilitate analysis (home, home with physical therapy, death, other (skilled nursing facility, long-term care, long-term acute care, acute rehabilitation facility, hospice)). ICU length of stay, inpatient length of stay, and duration of invasive MV were assessed as continuous variables measured in days.

The outcome of subjective symptom improvement measured at 28 days was collapsed to facilitate analysis as follows: better (“Great, I’m back to baseline,” “Much better, but not quite 100%,” “Okay, but better than before”) and same/worse (“Same as during hospitalization,” “Worse than during hospitalization”). Data entered for this variable in error for patients deceased at 28 days were removed.

Additional variables of interest include demographic and baseline clinical characteristics. The following data were collected: age in years, ethnicity (Hispanic, non-Hispanic, unknown), race (American Indian/Alaska Native, Asian, Black/African American, White/multiracial, unknown), gender (female, male), steroid initiation prior to the study (yes, no, unknown), body mass index (BMI), dexamethasone dose, pre-enrollment nadir oxygen saturation, oxygen required on enrollment (yes, no), oxygen modality on enrollment (nasal cannula, venturi mask, non-rebreather mask, high-flow nasal cannula, non-invasive ventilation, MV, unknown), Charlson Comorbidity Index, and 4C mortality score, a model that has been validated to predict in-hospital mortality in patients admitted with COVID-19 [28].

Statistical analysis

Demographic characteristics and baseline clinical data were summarized descriptively overall and by treatment arm. Specifically, categorical variables were summarized using frequencies and percentages, and continuous variables were summarized using means and standard deviations. No inferential comparisons between treatment arms were made with respect to demographic and baseline clinical data.

The primary outcome, all-cause mortality at 28 days, was compared across the two treatment arms using the chi-square test. All binary and categorical secondary and safety outcomes, including ICU admission, higher oxygen supplementation, MV, ECMO, tracheostomy, discharge disposition, oxygen supplementation on discharge, secondary bacterial or fungal infection, and clinically significant hyperglycemia, were compared across the two treatment arms using the chi-square test or Fisher’s exact test (if expected counts were <5 in more than 20% of cells).

All continuous secondary outcomes (ICU length of stay, inpatient length of stay, and duration of MV) were compared across the two treatment arms using the non-parametric Wilcoxon rank sum test (assumptions for the two-sample t-test were violated).

Sensitivity analyses were conducted for the secondary outcomes of MV and duration of MV by defining MV only as having escalation to MV. In these scenarios, patients on MV at enrollment were classified as having no escalation to ventilation. Analyses were rerun using these classifications to assess how robust the results were to these changes.

The intention-to-treat principle was employed (i.e., all randomized patients were analyzed in the groups to which they were randomly assigned, regardless of the intervention they truly received). A p-value of <0.05 was considered statistically significant for the primary outcome, and a Bonferroni-adjusted p-value of <0.004 was considered statistically significant for secondary and safety outcomes (0.05/13 secondary/safety outcomes). Study data were managed and collected using REDCap [26,27] electronic data capture tools hosted at Northwell Health, and all statistical analyses were performed using SAS Studio version 3.8 (SAS Institute Inc., Cary, NC).

## Results

**Figure 1:**
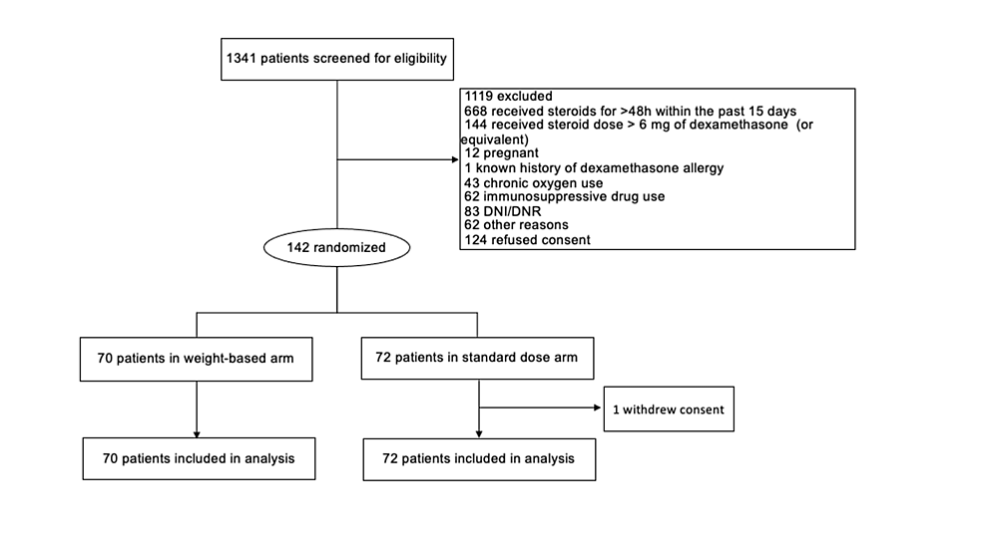
Screening and randomization of patients in the ROIDS-Dose trial DNI: do not intubate; DNR: do not resuscitate.

Between March 10, 2021, and December 28, 2021, 1341 patients were screened and 142 were enrolled. Seventy patients were randomized to the intervention group to receive the weight-based dexamethasone dose, and 72 patients were randomized to the control group to receive the standard dexamethasone dose (Figure 1).

Patient characteristics at baseline were similar in the two groups (Table 1). The mean age of the study participants was 56 ± 15 years. Ninety-nine patients were male (69.7%), 29 (20.4%) were Hispanic, and 51 (35.9%) were Black or African American. The mean BMI was 32.6 ± 9.4 kg/m2. The mean nadir oxygen saturation prior to enrollment was 84 ± 9%. At the time of enrollment, 91 (64%) patients were receiving oxygen supplementation via nasal cannula, two (1.4%) via venturi mask, 14 (9.86%) via non-rebreather mask, 25 (17.6%) via high-flow nasal cannula, five (3.5%) via non-invasive ventilation, and five (3.5%) via invasive MV.

**Table 1:** Baseline characteristics * n = 68; ^†^ n = 71.

	Treatment arm: dexamethasone 0.2 mg/kg (n = 70)	Control arm: dexamethasone 6 mg (n = 72)
Age, mean (SD), y	55.46 (15.11)	57.22 (15.18)
Ethnicity, No. (%)		
Hispanic	14 (20.00)	15 (20.83)
Non-Hispanic	55 (78.57)	56 (77.78)
Unknown	1 (1.43)	1 (1.39)
Race, No. (%)		
American Indian/Alaska Native	0 (0.00)	1 (1.39)
Asian	9 (12.86)	9 (12.50)
Black/African American	26 (37.14)	25 (34.72)
White	25 (35.71)	27 (37.50)
Multiracial	10 (14.29)	10 (13.89)
Gender, No. (%)		
Female	20 (28.57)	23 (31.94)
Male	50 (71.43)	49 (68.06)
Steroid initiation prior to the study, No. (%)	66 (94.29)	68 (94.44)
BMI, mean (SD)	33.57(9.84)^*^	31.72(8.99)^†^
Dexamethasone dose, mean (SD)	17.49 (2.66)	6.00 (0.00)
Pre-enrollment nadir oxygen saturation, mean (SD)	84.43 (7.96)	83.92 (10.72)
Oxygen required on enrollment, No. (%)	70 (100.00)	72 (100.00)
Oxygen modality on enrollment, No. (%)		
Nasal cannula	49 (70.00)	42 (58.33)
Venturi mask	1 (1.43)	1 (1.39)
Non-rebreather mask	5 (7.14)	9 (12.50)
High-flow nasal cannula	11 (15.71)	14 (19.44)
Non-invasive ventilation	3 (4.29)	2 (2.78)
Mechanical ventilation	1 (1.43)	4 (5.56)
Charlson Comorbidity Index, mean (SD)	1.90 (1.95)	2.18 (1.92)
4C mortality score, mean (SD)	7.86 (3.52)	8.67 (3.69)
Other treatments for COVID-19, No. (%)		
Tocilizumab	17 (24.29)	18 (25.00)
Remdesivir	65 (92.86)	65 (90.28)
Monoclonal antibodies	2 (2.86)	0 (0)
Any COVID-19 vaccination	9 (12.86)	8 (11.11)

Both groups had similar baseline comorbidities with a mean Charlson Comorbidity Index of 1.90 ± 1.95 in the intervention arm vs. 2.18 ± 1.92 in the control arm. The 4C mortality score was also similar in both groups (7.86 ± 3.52 in the intervention group vs. 8.67 ± 3.69 in the control group).

The use of other therapies for COVID-19 was similar between the two groups. A total of 130 (91.55%) patients received remdesivir: 65 (92.86%) in the intervention arm vs. 65 (90.28%) in the control group. Thirty-five (24.65%) patients received tocilizumab: 17 (24.29%) in the intervention group and 18 (25%) in the control arm. Two (2.86%) patients in the intervention group and none in the control group received anti-SARS-CoV-2 monoclonal antibodies in the outpatient setting prior to the hospitalization. A total of 17 (11.97%) patients had received at least one dose of the COVID-19 vaccine: nine (12.86%) in the intervention arm and eight (11.11%) in the control group.

The average dose of dexamethasone in the intervention group was 17.49 ± 2.66 mg while all controls received 6 mg. All patients received the dexamethasone dose assigned at the time of randomization for 10 days or until discharge, without crossover between the groups.

Follow-up was completed for all patients 28 days after enrollment. One patient from the control group withdrew consent from the study but available data were analyzed as per the intention-to-treat protocol.

There was no statistically significant difference in the primary outcome of 28-day all-cause mortality between the two groups (Table 2), with deaths in 12 of 70 patients (17.14%) in the intervention group vs. 15 of 72 patients (20.83%) in the control group (p = 0.58).

**Table 2:** Study outcomes * P-value cutoff for primary outcome = 0.05. P-value cutoff for secondary/safety outcomes = 0.004 after adjusting for multiple comparisons. ^†^ Among patients admitted to the ICU. ^‡^ Among patients with any mechanical ventilation. ^§^ Among patients with escalation to mechanical ventilation. ^ll^ Among patients discharged alive. ^¶^ Among patients alive at 28 days with a completed response.

	Treatment arm: dexamethasone 0.2 mg/kg (n = 70)	Control arm: dexamethasone 6 mg (n = 72)	P-value^*^
Primary outcome			
28-day mortality, No. (%)	12 (17.14)	15 (20.83)	0.58
Secondary outcomes			
ICU admission, No. (%)	12 (17.14)	27 (37.50)	0.007
ICU length of stay, days,mean (SD)^†^	11.33 (8.85)	15.33 (11.28)	0.30
Inpatient length of stay, days, mean (SD)	11.56 (8.88)	14.83 (17.00)	0.88
Higher oxygen supplementation, No. (%)	31 (44.29)	32 (44.44)	0.98
Any invasive mechanical ventilation, No. (%)	11 (15.71)	23 (31.94)	0.02
Escalation to invasive mechanical ventilation, No. (%)^‡^	10 (14.29)	19 (26.39)	0.07
Duration of invasive mechanical ventilation, days, mean (SD)^§^	10.90 (8.88)	19.95 (16.14)	0.14
Extracorporeal membrane oxygenation (ECMO), No. (%)	2 (2.86)	3 (4.17)	>0.99
Tracheostomy, No. (%)	1 (1.43)	5 (6.94)	0.21
Discharge disposition, No. (%)			0.81
Home	42 (60.00)	40 (55.56)	
Home with physical therapy	11 (15.71)	10 (13.89)	
Other	5 (7.14)	5 (6.94)	
Expired	12 (17.14)	17 (23.61)	
Oxygen supplementation on discharge, No. (%)^ll^	27 (46.55)	25 (45.45)	0.91
Subjective symptoms at 28 days^¶^			0.65
Better	44 (95.65)	33 (91.67)	
Same/worse	2 (4.35)	3 (8.33)	
Safety outcomes			
Secondary bacterial or fungal infection, No. (%)	9 (12.86)	19 (26.39)	0.04
Clinically significant hyperglycemia, No. (%)	4 (5.71)	5 (6.94)	>0.99

There were no statistically significant differences between the secondary and safety outcomes between the two groups (Table 2).

## Discussion

The ROIDS-Dose trial did not demonstrate a statistically significant difference in the primary outcome of mortality at 28-days between the dexamethasone 6 mg and the weight-based dexamethasone groups.

There were fewer patients requiring MV in the intervention group compared to the control group (15.71% vs. 31.94%; p = 0.02). However, there were fewer patients requiring MV at the time of enrollment in the intervention arm, and there was no statistically significant difference between the groups in escalation to MV (14.29% vs. 26.39%; p = 0.07).

One limitation of the study was that it was not blinded. Although a double-blinded study would have been desirable, an urgent need for additional data regarding effective therapies during the pandemic necessitated an expedited and more pragmatic trial design. Another limitation is the small sample size, which may have reduced the ability to detect statistically significant differences among some of the secondary outcomes. In addition, data were not collected regarding the timing of initiation of corticosteroid therapy in relation to symptom onset. Initiation of dexamethasone as early as possible may be an important metric in treatment if the goal of corticosteroid therapy is to blunt the overwhelming inflammatory response before a patient’s condition progresses to the point of clinical acute respiratory distress syndrome and fibrotic lung disease. Furthermore, selecting a maximum dose of 20 mg in the weight-based dexamethasone group may have affected the results, as 14 patients received less than 0.2 mg/kg based on the upper limit restriction of the study design. Finally, as the standard of care for the treatment of COVID-19 changed over time, those changes may have affected patient care in undetectable ways, affecting the results of the trial.

The trial does have several strengths. The pragmatic design and inclusion of a diverse patient population across community and tertiary hospitals increase its external validity. The investigation of weight-based, rather than fixed-dose dexamethasone, has not been previously studied in COVID-19 and represents an attempt to improve a pre-existing and widely available therapy. Though the ROIDS-Dose trial did not demonstrate differences in outcomes, the negative findings remain clinically useful and contribute new data to the existing literature regarding dexamethasone dosing in severe COVID-19. Finally, the study draws attention to several other potential areas of investigation regarding the use of corticosteroids for the treatment of COVID-19 pneumonia. The study was not powered to detect a difference in outcomes between subgroups. It would be of clinical interest to further evaluate if weight-based dosing of steroids would be beneficial in patients with obesity, given its high prevalence in the United States and worse clinical outcomes in obese patients with COVID-19 [29].

## Conclusions

Among patients with COVID-19 requiring oxygen supplementation, the use of weight-based dosing of dexamethasone was not superior to the standard dose in reducing the primary outcome of all-cause mortality at 28 days.

## References

[1] Pan H, Peto R, Henao-Restrepo AM (2021). Repurposed antiviral drugs for COVID-19 — interim WHO Solidarity Trial results. N Engl J Med.

[2] RECOVERY Collaborative Group (2020). Lopinavir-ritonavir in patients admitted to hospital with COVID-19 (RECOVERY): a randomised, controlled, open-label, platform trial. Lancet.

[3] Horby P, Mafham M, Linsell L (2020). Effect of hydroxychloroquine in hospitalized patients with COVID-19. N Engl J Med.

[4] Gordon AC, Mouncey PR, Al-Beidh F (2021). Interleukin-6 receptor antagonists in critically ill patients with COVID-19. N Engl J Med.

[5] Simonovich VA, Burgos Pratx LD, Scibona P (2021). A randomized trial of convalescent plasma in COVID-19 severe pneumonia. N Engl J Med.

[6] RECOVERY Collaborative Group (2022). Casirivimab and imdevimab in patients admitted to hospital with COVID-19 (RECOVERY): a randomised, controlled, open-label, platform trial. Lancet.

[7] Shi Y, Wang Y, Shao C (2020). COVID-19 infection: the perspectives on immune responses. Cell Death Differ.

[8] Wiersinga WJ, Rhodes A, Cheng AC, Peacock SJ, Prescott HC (2020). Pathophysiology, transmission, diagnosis, and treatment of coronavirus disease 2019 (COVID-19): a review. JAMA.

[9] Beigel JH, Tomashek KM, Dodd LE (2020). Remdesivir for the treatment of COVID-19 — final report. N Engl J Med.

[10] Hammond J, Leister-Tebbe H, Gardner A (2022). Oral nirmatrelvir for high-risk, nonhospitalized adults with COVID-19. N Engl J Med.

[11] Barnes PJ (2006). How corticosteroids control inflammation: Quintiles Prize Lecture 2005. Br J Pharmacol.

[12] Williams DM (2018). Clinical pharmacology of corticosteroids. Respir Care.

[13] Villar J, Ferrando C, Martinez D (2020). Dexamethasone treatment for the acute respiratory distress syndrome: a multicentre, randomised controlled trial. Lancet Respir Med.

[14] Russell CD, Millar JE, Baillie JK (2020). Clinical evidence does not support corticosteroid treatment for 2019-nCoV lung injury. Lancet.

[15] Horby P, Lim WS, Emberson JR (2021). Dexamethasone in hospitalized patients with COVID-19. N Engl J Med.

[16] Bhimraj A, Morgan RL, Shumaker AH (2020). Infectious Diseases Society of America guidelines on the treatment and management of patients with coronavirus disease 2019 (COVID-19). Clin Infect Dis.

[17] Agarwal A, Rochwerg B, Lamontagne F (2020). A living WHO guideline on drugs for COVID-19. BMJ.

[18] (2022). National Institutes of Health. Coronavirus disease 2019 (COVID-19) treatment guidelines. https://www.covid19treatmentguidelines.nih.gov/.

[19] Tomazini BM, Maia IS, Cavalcanti AB (2020). Effect of dexamethasone on days alive and ventilator-free in patients with moderate or severe acute respiratory distress syndrome and COVID-19: the CoDEX randomized clinical trial. JAMA.

[20] Edalatifard M, Akhtari M, Salehi M (2020). Intravenous methylprednisolone pulse as a treatment for hospitalised severe COVID-19 patients: results from a randomised controlled clinical trial. Eur Respir J.

[21] Sterne JA, Murthy S, Diaz JV (2020). Association between administration of systemic corticosteroids and mortality among critically ill patients with COVID-19: a meta-analysis. JAMA.

[22] Munch MW, Myatra SN, Vijayaraghavan BK (2021). Effect of 12 mg vs 6 mg of dexamethasone on the number of days alive without life support in adults with COVID-19 and severe hypoxemia: the COVID STEROID 2 randomized trial. JAMA.

[23] Taboada M, Rodríguez N, Varela PM (2022). Effect of high versus low dose of dexamethasone on clinical worsening in patients hospitalised with moderate or severe COVID-19 pneumonia: an open-label, randomised clinical trial. Eur Respir J.

[24] Jang HJ, Yong SH, Leem AY (2021). Corticosteroid responsiveness in patients with acute exacerbation of interstitial lung disease admitted to the emergency department. Sci Rep.

[25] Janahi IA, Rehman A, Baloch NUA (2017). Corticosteroids and their use in respiratory disorders. Corticosteroids.

[26] Harris PA, Taylor R, Minor BL (2019). The REDCap consortium: building an international community of software platform partners. J Biomed Inform.

[27] Harris PA, Taylor R, Thielke R, Payne J, Gonzalez N, Conde JG (2009). Research Electronic Data Capture (REDCap)—a metadata-driven methodology and workflow process for providing translational research informatics support. J Biomed Inform.

[28] Knight SR, Ho A, Pius R (2020). Risk stratification of patients admitted to hospital with COVID-19 using the ISARIC WHO Clinical Characterisation Protocol: development and validation of the 4C mortality score. BMJ.

[29] Richardson S, Hirsch JS, Narasimhan M (2020). Presenting characteristics, comorbidities, and outcomes among 5700 patients hospitalized with COVID-19 in the New York City area. JAMA.

